# Novel lactotransferrin-derived synthetic peptides suppress cariogenic bacteria *in vitro* and arrest dental caries *in vivo* [Novel lactotransferrin-derived anticaries peptides]

**DOI:** 10.1080/20002297.2021.1943999

**Published:** 2021-06-20

**Authors:** Junyuan Luo, Zening Feng, Wentao Jiang, Xuelian Jiang, Yue Chen, Xiaohui Lv, Linglin Zhang

**Affiliations:** a State Key Laboratory of Oral Diseases and National Clinical Research Centre for Oral Diseases, Sichuan University, Chengdu, China; b Department of Cariology and Endodontics, West China Hospital of Stomatology, Sichuan University, Chengdu, China; c Hospital of Stomatology, Guangdong Provincial Key Laboratory of Stomatology, Guanghua School of Stomatology, Sun Yat-sen University, Guangzhou, China

**Keywords:** Antimicrobial peptide, biofilm, dental caries, lactotransferrin, rat, *Streptococcus mutans*

## Abstract

**Objectives**: The aim of the study was to design and synthesise novel lactotransferrin-derived antimicrobial peptides (AMPs) with enhanced antibacterial activity against cariogenic bacteria.

**Methods**: We obtained the LF-1 (WKLLRKAWKLLRKA) and LF-2 (GKLIWKLLRKAWKLLRKA) AMPs, based on the N-terminal functional sequence of lactotransferrin, and characterised their physicochemical properties and secondary structure. Their antibacterial activity against caries-associated bacteria was evaluated using bacterial susceptibility and time-killing assays, as well as transmission electron microscopy (TEM). The antibiofilm activity against *Streptococcus mutans* biofilms was determined using biofilm susceptibility assays and confocal laser scanning microscopy (CLSM). A rodent model of dental caries was adopted to evaluate their anticaries effectiveness *in vivo*.

**Results**: Both peptides possessed an α-helical structure with excellent amphipathicity. LF-1 was effective against *S. mutans* and *Actinomyces* species, whereas LF-2 showed more potent antibacterial activity than LF-1 against a broader spectrum of tested strains. Both peptides inhibited the formation of *S. mutans* biofilm starting at 8 μmol/L and exerted effective eradication of *S. mutans* in preformed biofilms. Both peptides exhibited satisfactory biocompatibility and exerted significant anticaries effects in a rodent model.

**Conclusions:** Both lactotransferrin-derived peptides displayed strong antimicrobial activity against cariogenic bacteria and *S. mutans* biofilm *in vitro* and effectively inhibited dental caries *in vivo*.

## Introduction

Dental caries is among the most widespread chronic infectious diseases worldwide, causing progressive destruction of dental hard tissues and imposing huge burdens on public healthcare systems [1]. Cariogenic bacteria in oral biofilm digest dietary carbohydrates and produce organic acids, resulting in the demineralisation of tooth surfaces [2]. In particular, *Streptococcus mutans* has been highlighted as a principal cariogenic bacterium due to its high acidogenic capacity, potent aciduricity, and ability to adhere to tooth surfaces [3]. In addition, *S. mutans* can also generate exopolysaccharides, reinforcing the aggregation of oral bacteria and thus stabilising the structure of dental biofilm [4]. Therefore, control and suppression of *S. mutans* growth and cariogenic biofilm formation are essential approaches to preventing dental caries [5].

Antimicrobial peptides (AMPs) have attracted much attention in recent years, and have become promising anticaries agents due to their ability to exert potent and effective antimicrobial effects against cariogenic bacteria and biofilms [6,7]. Among the most representative AMPs, α-helical peptides can exert their antibacterial action via membrane disruption due to their hydrophobic-cationic (amphiphilic) structure that endows them with an α-helical fold when interacting with membrane lipid bilayers [8]. Considering their therapeutic potential in treating infectious diseases, many natural and synthetic AMPs, such as human β-defensins, LL37 [9], and GH12 [10,11], have been demonstrated to inhibit the growth of cariogenic bacteria and arrest dental caries. Nevertheless, only a small number of natural AMPs have been reported to treat dental caries [12], perhaps because of the relatively weak antibacterial activity of most of them. To overcome this issue, a practical approach could be to design novel mimetics with improved antibacterial activity based on the functional sequences of natural antimicrobial proteins and peptides [13]. This inspired us to extract template sequences from native anticaries proteins and design sequence alterations to obtain promising functional AMPs for treating dental caries.

Lactotransferrin, a natural iron-binding glycoprotein with antimicrobial activity, is a constituent of saliva and the acquired pellicle [14,15]. Proteomic analysis has demonstrated that the levels of lactotransferrin were dramatically elevated in the acquired pellicle of caries-susceptible subjects [16], revealing its important role as a caries-associated protein. Although lactotransferrin can exert bactericidal action against *S. mutans* and mediate bacterial agglutination, markedly high concentrations are required to effectively perform an anticaries function [17]. The successful identification of several antimicrobial functional domains at the N-terminus of lactotransferrin has inspired the development of synthetic AMPs with a certain degree of antibacterial properties [18]. However, few studies have explored the direct effects of lactotransferrin-derived peptides on cariogenic bacteria and dental caries. Thus, developing peptides derived from the functional antimicrobial region of lactotransferrin to achieve improved anticaries effectiveness warrants investigation.

A 17-residue sequence from the N-terminus of lactotransferrin (named lactoferrampin) has attracted the attention of researchers due to its notable amphiphilic characteristics, such as an N-terminal helix and a flexible cationic C-terminus [19]. Furthermore, the N-terminal portion of lactoferrampin has been reported to be the crucial domain responsible for its antimicrobial activity [20], suggesting that its functional sequence could be used to design and obtain more satisfactory lactotransferrin-derived peptides.

Therefore, based on the N-terminal functional domain of lactoferrampin, the aim of the study was to design and synthesise novel lactotransferrin-derived AMPs and confirm their antimicrobial activity and anticaries effects. Our null hypothesis was defined as no antimicrobial activity against cariogenic bacteria and *S. mutans* biofilm *in vitro* and no anticaries effects *in vivo*. To the best of our knowledge, this is the first attempt to modify the functional sequence of lactoferrampin and synthesise lactotransferrin-derived AMPs applied in the study of dental caries, in contrast with direct sequence interception in previous studies.

## Materials and methods

### Peptide synthesis and sequence analysis

The LF-1 (WKLLRKAWKLLRKA), LF-2 (GKLIWKLLRKAWKLLRKA), template (WKLLRKA), and parent lactoferrampin (WKLLSKAQEKFGKNKSR) peptides were synthesised using standard 9-fluorenylmethoxycarbonyl (Fmoc) solid-phase protocols [21] and verified by mass spectrometry (3200 Q TRAP, Thermo Fisher Scientific, Waltham, MA, USA). Peptides were then purified by reverse-phase high-performance liquid chromatography (RP-HPLC; Thermo Fisher Scientific) to > 95% and stored at −20°C until use.

The physicochemical properties of peptides were analysed according to the following parameters [22]: hydrophobic moment (μH) and helical-wheel diagrams were determined at Heliquest (https://heliquest.ipmc.cnrs.fr/); molecular weight (MW) and net charge at pH 7 were calculated using the Peptide Property Calculator (http://www.pepcalc.com/ppc.php); and grand average hydropathy (GRAVY) was determined using the GRAVY Calculator (www.gravy-calculator.de).

### Circular dichroism (CD) spectrum analysis

Peptides were dissolved to a final concentration of 100 μmol/L (μM) in 20 mmol/L (mM) phosphate-buffered saline (PBS) alone or supplemented with 25 mM sodium dodecyl sulfonate (SDS) [23]. The chirality of peptides was determined using a CD spectrometer (J-1500, JASCO, Tokyo, Japan) with a quartz cell of 1 mm optical path length. Wavelengths were scanned from 197 to 250 nm at 1 nm intervals and a rate of 50 nm/min. CD spectra were obtained from an average of 10 scans and expressed as the mean residue ellipticity θ (deg × M^–1^ × m^–1^). The α-helical ratios of peptides were calculated using the program CONTINLL in CDPro (http://lamar.colostate.edu/∼sreeram/CDPro/main.html) [24].

### Tryptophan fluorescence measurement

Large unilamellar vesicles (LUVs) of dimyristoyl phosphatidylglycerole (DMPG; TCI, Tokyo, Japan) were prepared following the previously described protocol [20]. Peptides were dissolved to a final concentration of 10 μM in 10 mM HEPES buffer (containing 100 mM NaCl, pH 7.4) alone or supplemented with 0.5 mg/mL LUVs. The analysis was performed on a multi-mode microplate reader (SpectraMax iD5, Molecular Devices, San Jose, CA, USA) at an excitation wavelength of 280 nm. Emission spectra were recorded from 320 to 450 nm with slit widths of 5 nm and obtained from an average of five scans [19].

### Bacterial inoculation and culture

All tested bacterial strains were obtained from our laboratory. *S. mutans* UA159, *Streptococcus sanguinis* ATCC10,556, *Streptococcus gordonii* ATCC 10,558, *Streptococcus mitis* ATCC 6,249, *Streptococcus salivarius* ATCC 7,073, *Streptococcus sobrinus* ATCC 33,478, *Actinomyces viscosus* ATCC 15,987, and *Actinomyces naeslundii* ATCC 12,104 were cultured in brain-heart infusion broth (BHI; Oxoid, Basingstoke, Hampshire, UK). *Lactobacillus acidophilus* ATCC 4,356, *Lactobacillus casei* ATCC 393, and *Lactobacillus fermentium* ATCC 14,931 were grown in de Man, Rogosa, and Sharpe broth (MRS; Oxoid). All strains were incubated anaerobically (85% N_2_, 10% H_2_, and 5% CO_2_) at 37°C [25].

### Bacterial susceptibility assay

Minimal inhibitory concentration (MIC) and minimal bactericidal concentration (MBC) were determined using the modified broth microdilution method [10]. Briefly, 2-fold serial dilutions of peptides were prepared with bacterial suspensions in 96-well plates. Final peptide concentrations ranged from 256 to 1 μM, and the final bacterial concentration was approximately 1 × 10^6^ colony-forming units (CFU)/mL. Negative and blank controls were also prepared using sterilised distilled deionised water (DDW) and only broths, respectively. After anaerobic incubation for 24 h at 37°C, the optical density was measured at 600 nm (OD_600_) using a microplate spectrophotometer (Multiskan GO, Thermo Fisher Scientific). MIC was defined as the lowest concentration with no difference in OD_600_ compared with the blank control. Aliquots (100 μL) of bacterial suspensions from wells with peptide concentrations equal and above the MIC were cultured on broth agar medium anaerobically for 48 h at 37°C. MBC was defined as the lowest concentration with no bacterial colony growth. MIC and MBC values were represented as the mean ± standard deviation (SD) of five independent experiments.

### Transmission electron microscopy (TEM) observation of bacterial morphology

The *S. mutans, L. acidophilus*, and *A. viscosus* bacteria were chosen to analyse the effects of peptides on planktonic bacterial morphology, as previously described [26]. Bacterial suspensions in the logarithmic phase (approximately 1 × 10^9^ CFU/mL) were treated with 64 μM LF-1 and LF-2, followed by anaerobic incubation for 24 h at 37°C. Treated cells were collected by centrifugation at 4,500 *g* for 5 min at 4°C, washed twice with PBS, fixed with 2.5% (v/v) glutaraldehyde overnight at 4°C, and post-fixed with 1% osmium tetroxide (Leica, Wetzlar, Germany) for 3 h. Fixed cells were subsequently dehydrated using an acetone gradient (30, 50, 70, 80, 90, 95, and 100%). Dehydrated samples were embedded in epoxy resin, sliced into thin sections (approximately 50 nm), and stained first with uranyl acetate (Merck, Darmstadt, Germany) for 15 min, and then with lead citrate (Sigma-Aldrich, St. Louis, MO, USA) for 2 min at 25°C. Finally, the stained sections were viewed using TEM (JEM-1400 PLUS, JEOL, Tokyo, Japan) and their images captured.

### Time-killing assay

A time-killing assay was conducted to validate the bactericidal efficiency of peptides against two typical cariogenic bacteria, *S. mutans* and *A. viscosus* [27]. Briefly, peptide concentrations of 1-, 2-, and 4-fold MBC were added to bacterial suspensions (1 × 10^6^ CFU/mL), which were incubated anaerobically for 0, 1, 5, 10, 20, 30, 60, and 120 min at 37°C. DDW was used as the negative control. At each time point, aliquots (100 μL) were collected, diluted, and spread on BHI agar, and the resulting bacterial colonies were counted after anaerobic incubation for 48 h at 37°C. Time-killing curves were plotted using data from three independent experiments.

### Biofilm susceptibility assay

The effects of peptides on *S. mutans* biofilm formation were examined using the modified microdilution method [28]. Briefly, a culture of *S. mutans* was diluted in brain heart infusion-sucrose broth (BHIS; BHI containing 1% sucrose) to a final concentration of 1 × 10^6^ CFU/mL, and peptides were added at final concentrations ranging from 64 to 1 μM. After anaerobic incubation for 24 h at 37°C, biofilms were washed with PBS to remove planktonic bacteria. Biofilms were fixed with methanol for 15 min and stained with 0.1% (w/v) crystal violet for 5 min. The excess crystal violet was washed away by PBS, and the bound dye was resolubilised with 33% (v/v) glacial acetic acid. Biofilm biomass was quantified by measuring absorbance at 595 nm using a microplate spectrophotometer. MBIC_90_ was defined as the minimal peptide concentration to inhibit ≥ 90% biofilm formation.

Subsequently, the eradicative effect of peptides on preformed *S. mutans* biofilms was examined [29]. Bacterial suspensions (final concentration of 1 × 10^6^ CFU/mL) were incubated anaerobically in 24-well plates for 24 h at 37°C to form mature biofilms. Biofilms were washed with PBS to remove non-adherent cells. Peptides were added to biofilms at concentrations ranging from 64 to 1 μM in fresh BHIS broth, followed by anaerobic incubation for 24 h at 37°C. Biofilms were then scraped into sterile tubes and resuspended in PBS. Biofilm suspensions were serially diluted, and aliquots (100 μL) were spread on BHI agar and incubated anaerobically for 48 h at 37°C. Resulting bacterial colonies were counted to determine the biomass in the preformed biofilms.

### Confocal laser scanning microscopy (CLSM) observation

Bacterial suspensions were incubated in glass-bottom dishes to obtain 1-d preformed biofilms, to which peptides were added at a final concentration of 64 μM [30]. PBS was used as the control. After anaerobic incubation for 24 h at 37°C, biofilms were stained using the LIVE/DEAD BacLight Bacterial Viability Kit (Invitrogen, Carlsbad, CA, USA) following the manufacturer’s instructions. Live cells were stained with SYTO 9 (green fluorescence), while dead cells were stained with propidium iodide (PI; red fluorescence). A CLSM (FV-1000, Olympus, Tokyo, Japan) equipped with a 40× objective lens was used to observe the labelled biofilms. Three-dimensional reconstructions of the biofilms were performed using the Imaris version 7.4.2 cell imaging software (Bitplane, Zürich, Switzerland), and biomass quantification was conducted using the COMSTAT software tool (http://www.imageanalysis.dk).

### Haemolysis assay

The haemolysis assay was approved by the ethics committee and conducted as previously described, with some modifications [31]. Sheep erythrocytes (Lonza, Basel, Switzerland) were collected by centrifugation at 75 *g* for 15 min at 4°C and washed with PBS until the supernatant was clear. Cell suspensions were diluted in PBS and normalised to a haemoglobin concentration of 2 mM. Aliquots (100 μL) of cell dilutions were added to 96-well plates supplemented with equal volumes of peptides in PBS. Each peptide was tested at concentrations ranging from 256 to 1 μM. 0.1% Triton X-100 was used as positive control, whereas PBS was used as negative control. The plates were incubated for 60 min at 37°C, and then centrifuged at 550 *g* for 5 min at 4°C. The absorbance of the supernatant was measured at 450 nm using a microplate spectrophotometer.

### In vivo animal experiment

The ethics committee approved the animal experiment, which was performed using a well-established rodent model of dental caries [32]. All experimental procedures strictly conformed to the ARRIVE guidelines [33] and were carried out following the U.K. Animals Act, 1986 [34] and associated guidelines. Briefly, 42 male, specific pathogen-free (SPF) Sprague-Dawley rats aged 21 days were purchased from the Animal Experimental Centre. Rats were fed a regular diet and water containing penicillin G sodium (4,000 U/mL) for 3 d to reduce the microbial load [35]. Subsequently, rats were orally inoculated with an actively growing *S. mutans* UA159 culture, and *S. mutans* colonisation of the oral cavity was confirmed by plating on mitis-salivarius bacitracin agar (MSB; BD Difco, Franklin Lakes, NJ, USA) [36]. Rats were randomly distributed into seven groups, and their oral cavities were treated topically three times daily as follows: (1) four treatment groups: 64 μM LF-1, 32 μM LF-1, 64 μM LF-2, and 32 μM LF-2; (2) two positive controls: 1,000 ppm sodium fluoride (NaF; Sigma-Aldrich) and 0.12% chlorhexidine (CHX; Sigma-Aldrich); (3) negative control: DDW. Rats were then fed a cariogenic Keyes 2000 diet and water containing 5% sucrose ad libitum for 5 wk [35]. All rats were weighed weekly, and their physical appearance was noted daily.

At the end of the experimental period, rats were euthanised by CO_2_ asphyxiation, and the jaws and oral soft tissues were surgically dissected and aseptically obtained. The molars of the jaws were stained with 0.4% murexide (Sigma-Aldrich) for 12 h, and evaluated using the Keyes caries scoring system [37]. The oral soft tissues were fixed in 4% paraformaldehyde solution, stained with haematoxylin and eosin (H&E), and embedded in neutral resin. After slicing, samples were scanned and observed under an optical microscope (CX-33, Olympus) for image analysis [11].

### Statistical analysis

Statistical analysis was conducted via one-way analysis of variance (ANOVA) using the GraphPad Prism version 8.0 software (GraphPad Software, La Jolla, CA, USA). A p-value of < 0.05 was considered statistically significant.

## Results

### Sequence analysis and structural characterisation of peptides

**Figure 1.**
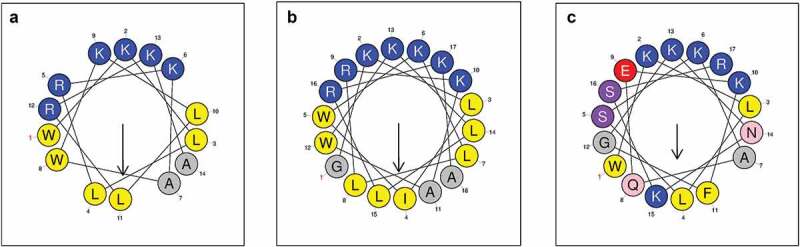
Helical wheel diagrams of LF-1 (**A**), LF-2 (**B**), and lactoferrampin (**C**). Arrows indicate the direction of the hydrophobic moment. Blue represents hydrophilic amino acid residues, whereas yellow represents hydrophobic amino acid residues. The hydrophobic faces of LF-1 and LF-2 are LLAALLWW and LLLAAILLGWW, respectively. No apparent amphiphilic interface exists in the sequence of lactoferrampin

The sequences and physicochemical parameters of peptides are presented in Table 1. We found that compared with the lactoferrampin parent peptide, LF-1 and LF-2 displayed higher hydrophobic moment (μH) values and net charge, but lower grand average of hydropathy (GRAVY) values. The helical wheel diagrams indicated that LF-1 and LF-2 possessed obvious amphiphilic interfaces with clearly separated hydrophilic and hydrophobic sectors, whereas the hydrophilic and hydrophobic amino acid residues were irregularly arranged in the lactoferrampin sequence (Figure 1).

**Table 1.** Amino acid sequences and physicochemical properties of peptides studied

Peptide	Sequence	MW	μH	Net charge	GRAVY
LF-1	WKLLRKAWKLLRKA	1810.28	0.679	+6	−0.54
LF-2	GKLIWKLLRKAWKLLRKA	2221.82	0.639	+7	−0.20
Lactoferrampin	WKLLSKAQEKFGKNKSR	2048.39	0.460	+5	−1.48

MW, molecular weight; μH, hydrophobic moment; GRAVY, grand average of hydropathy.

**Figure 2.**
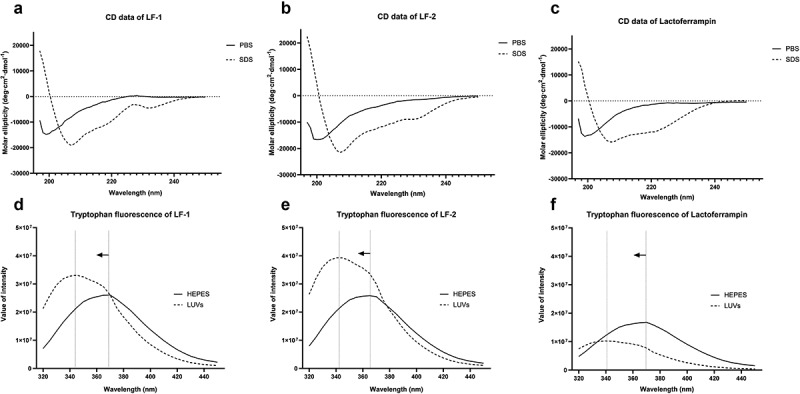
Circular dichroism and tryptophan fluorescence spectra of LF-1 (**A, D**), LF-2 (**B, E**), and lactoferrampin (**C, F**) in free solutions (PBS or HEPES) and membrane-mimetic solvents (SDS or LUVs). In the tryptophan fluorescence spectra, arrows indicate a blue shift of peak fluorescence towards a shorter wavelength

We observed that none of the peptides exhibited a typical secondary structure in PBS, but rather a strong α-helical structure in SDS buffer, with spectra showing characteristic double minima at 208 and 222 nm. Among peptides, LF-2 was shown to exhibited the strongest α-helical signals with an α-helical content of 65.0%, followed by LF-1 with 54.8% and lactoferrampin with 42.0% in SDS buffer (Figure 2A-C). Tryptophan fluorescence analysis revealed that the emission spectra of all peptides in LUVs buffer demonstrated a blue shift of peak fluorescence towards a shorter wavelength, indicating interaction with lipid membranes. We noticed that LF-1 and LF-2 displayed remarkably increased fluorescence intensity in LUVs buffer, with the intensity of LF-2 exceeding that of LF-1. In contrast, no apparent changes were observed in the fluorescence intensity of lactoferrampin both in HEPES and LUVs buffers (Figure 2D-E).”

### LF-1 and LF-2 possessed strong antibacterial activity against cariogenic bacteria

As shown in Table 2, LF-2 possessed a broader antibacterial spectrum and greater activity against caries-associated bacteria than LF-1. More specifically, LF-1 showed characteristic activity against *S. mutans* and *A. viscosus*, with MIC values of 8.00 μM and MBC values of 8.00–16.00 μM. However, we noticed that LF-1 displayed relatively weaker antibacterial activity against other tested bacteria, with MIC and MBC values ranging from 16.00 to 256.00 μM. Interestingly, LF-2 exerted more potent antibacterial activity against most tested strains with relatively low MIC and MBC values. In contrast, we found that both the template peptide and lactoferrampin did not possess antimicrobial properties under the experimental conditions, with MIC and MBC values exceeding 256.00 μM.

**Table 2.** Minimum inhibitory concentration and minimum bactericidal concentration of peptides on tested bacteria

Strains	LF-1		LF-2		Template	Lactoferrampin
	MIC	MBC	MIC	MBC	MIC/MBC	MIC/MBC
*S.* *mutans*	8.00 ± 0.00	16.00 ± 0.00	16.00 ± 0.00	32.00 ± 0.00		
*S.* *sanguinis*	128.00 ± 0.00	128.00 ± 0.00	16.00 ± 0.00	32.00 ± 0.00		
*S.* *gordonii*	89.60 ± 35.05	204.80 ± 70.11	32.00 ± 0.00	32.00 ± 0.00		
*S.* *salivarius*	16.00 ± 0.00	25.60 ± 8.76	11.20 ± 4.38	16.00 ± 0.00		
*S.* *sobrinus*	32.00 ± 0.00	64.00 ± 0.00	16.00 ± 0.00	32.00 ± 0.00		
*S.* *mitis*	>256.00		>256.00		>256.00	>256.00
*L.* *acidophilus*	64.00 ± 0.00	128.00 ± 0.00	8.00 ± 0.00	16.00 ± 0.00		
*L.* *fermentum*	64.00 ± 0.00	64.00 ± 0.00	8.00 ± 0.00	16.00 ± 0.00		
*L.* *casei*	>256.00		64.00 ± 0.00	128.00 ± 0.00		
*A.* *viscosus*	8.00 ± 0.00	8.00 ± 0.00	8.00 ± 0.00	8.00 ± 0.00		
*A.* *naeslundii*	8.00 ± 0.00	16.00 ± 0.00	8.00 ± 0.00	16.00 ± 0.00		

MIC, minimum inhibitory concentration (μmol/L); MBC, minimum bactericidal concentration (μmol/L). Data are presented as the mean ± SD, n = 5.

**Figure 3.**
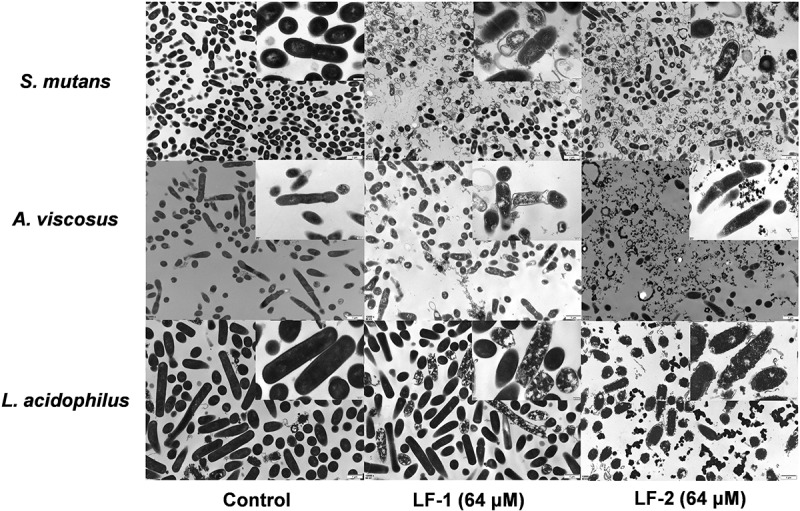
TEM observations of bacterial membrane and intracellular structure of *S. mutans, A. viscosus*, and *L. acidophilus* after treatment with 64 μM LF-1 and LF-2

Morphological changes in the bacterial membrane and intracellular structure induced by treatment with LF-1 and LF-2 are shown in Figure 3. As expected, untreated bacteria displayed an intact cell surface and dense intracellular structure with occasional cell lysis and debris. After exposure to 64 μM LF-1, we noticed that *S. mutans* and *A. viscosus* exhibited large-scale cell death, membranolysis, and cytoplasmic disorder, whereas *L. acidophilus* demonstrated relatively less cell lysis and cytoplasmic disorder. We also found that exposure to 64 μM LF-2 induced intensively destructive effects on the membrane and intracellular structure of all tested strains.

The results of the time-killing assay indicated that both LF-1 and LF-2 exerted strong bactericidal effects within 60 min, with higher peptide concentrations leading to more rapid reduction in the number of viable bacteria (Supplementary Figure 1). In particular, we observed that LF-1 eradicated *S. mutans* completely at 1-, 2-, and 4-fold MBC within 60, 60, and 5 min, respectively. Moreover, LF-2 fully eradicated *S. mutans* within 1 min at 1-fold MBC. Both LF-1 and LF-2 were shown to completely eradicated *A. viscosus* at only 1-fold MBC within 30 and 20 min, respectively.

### LF-1 and LF-2 inhibited formation of S. mutans biofilms

**Figure 4.**
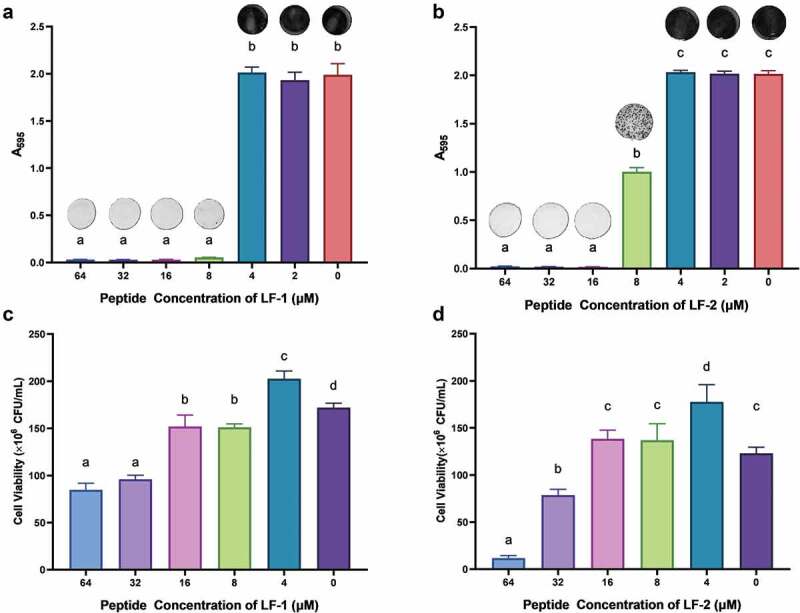
The inhibitory effect of LF-1 and LF-2 on the formation of *S. mutans* biofilm (**A, B**), as well as the eradication of 1-d preformed *S. mutans* biofilm by LF-1 and LF-2 (**C, D**) are illustrated by the absorbance at 595 nm and CFU number, respectively. Columns labelled with different superscript letters denote significant statistical differences among treatments (One-way ANOVA, p < 0.05). For the biofilm inhibition assay (**A, B**), representative images of stained biofilms treated with peptides at concentrations ranging from 64 to 0 μM are presented above the columns

Both LF-1 and LF-2 exerted strong inhibitory effects on the formation of *S. mutans* biofilm with MBIC_90_ values of 8 and 16 μM, respectively (Figure 4A-B). Further, we noticed that LF-2 began exerting its inhibitory effect at 8 μM with an inhibitory rate of approximately 50%. More specifically, LF-1 was shown to significantly reduced the bacterial content in 1-d preformed *S. mutans* biofilm starting at an effective concentration of 8 μM (p < 0.05), and killing approximately 50% of *S. mutans* within the biofilm at a concentration of 64 μM. Similarly, LF-2 induced significant bacterial eradication in the *S. mutans* biofilm at an effective concentration of 32 μM (p < 0.05), eventually killing over 90% of *S. mutans* within the biofilm at a concentration of 64 μM (Figure 4C-D).

**Figure 5.**
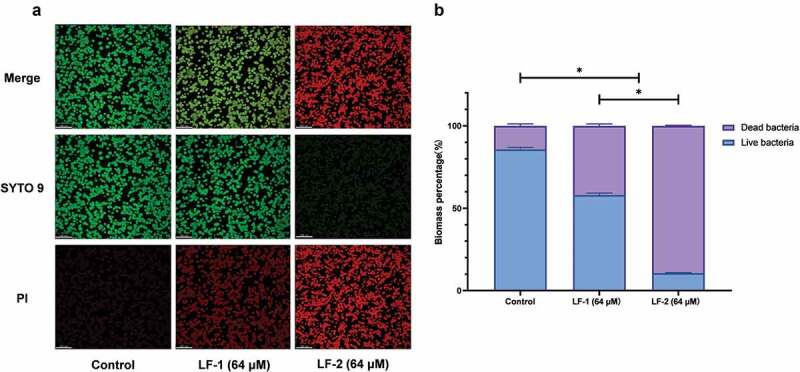
(**A**) Representative CLSM images of live, dead, and merged bacterial cells in preformed *S. mutans* biofilms treated with peptides for 24 h. Live cells are stained green with SYTO 9, whereas dead cells are stained red with propidium iodide (PI). (**B**) Biomass percentages of live and dead cells, calculated according to five randomly selected images. Data are presented as the mean ± SD (*p < 0.05)

Confocal laser scanning microscopy (CLSM) confirmed that LF-1 and LF-2 had an eradicative effect on *S. mutans* biofilm (Figure 5A). We observed that the *S. mutans* biofilm was stained mostly green in the control group (live cells), whereas the red area (dead cells) was increased, and its intensity was close to that of the green area after treatment with 64 μM LF-1. Moreover, we found that the *S. mutans* biofilm was stained almost completely red and exhibited extremely weak green fluorescence after treatment with 64 μM LF-2. The live/dead-cell ratio indicated that the percentage of dead cells in the *S. mutans* biofilm increased to 42.02 ± 1.19% and 89.38 ± 0.37% after treatment with 64 μM LF-1 and LF-2, respectively (Figure 5B).

### LF-1 displayed lower haemolytic toxicity than LF-2

We noticed that LF-1 did not demonstrate haemolytic toxicity towards erythrocytes, despite a slight incremental increase of the haemolysis ratio at 128 μM LF-1. In contrast, we found that the haemolytic toxicity of LF-2 was concentration-dependent and was dramatically increased at concentrations exceeding 64 μM. In particular, an LF-2 concentration exceeding 128 μM was shown to caused 50% haemolysis. In general, we concluded that LF-1 possessed much lower haemolytic toxicity than LF-2 (Supplementary Figure 2).

### LF-1 and LF-2 exerted significant anticaries effects and satisfactory biocompatibility in the rodent model

As shown in Table 3, the Keyes scores of smooth-surface and sulcal-surface caries were significantly reduced in the molars of rats following topical use of LF-1, LF-2, CHX, and NaF compared with those in the DDW group (p < 0.05). After treatment with 64 μM LF-2 and 1,000 ppm NaF, the Keyes scores for slight and moderate dentinal sulcal lesions were significantly lower than those of other groups (p < 0.05). In addition, the groups treated with 64 μM and 32 μM LF-1, 32 μM LF-2, and 0.12% CHX had similar Keyes scores for sulcal-surface caries, which surpassed those detected in the DDW group.

**Table 3.** Evaluation of Keyes caries scoring method on smooth and sulcal surfaces of rat molars compared across experimental groups

	Smooth surfaces		Sulcal surfaces			
	E	Ds	E	Ds	Dm	Dx
LF-1 (64 μM)	12.00 ± 2.37^a^	4.33 ± 1.37 ^c^	24.16 ± 1.60^d^	19.50 ± 1.64 ^g^	9.00 ± 1.26 ^j^	1.17 ± 0.98 ^m^
LF-1 (32 μM)	13.00 ± 1.67^a^	4.17 ± 1.17 ^c^	24.16 ± 1.47^d^	19.00 ± 1.90 ^g^	9.00 ± 1.41 ^j^	0.67 ± 0.82 ^m^
LF-2 (64 μM)	10.67 ± 1.86^a^	3.00 ± 0.89 ^c^	21.83 ± 1.72^e^	16.33 ± 1.97 ^h^	6.33 ± 0.82^k^	0.50 ± 0.84 ^m^
LF-2 (32 μM)	12.00 ± 1.26^a^	3.50 ± 1.05 ^c^	24.33 ± 1.21^d^	20.00 ± 0.89 ^g^	9.50 ± 1.38 ^j^	1.00 ± 0.89 ^m^
DDW	18.83 ± 1.17^b^	3.50 ± 1.38 ^c^	28.83 ± 0.75 ^f^	25.17 ± 0.75^i^	16.67 ± 1.63 ^l^	5.00 ± 0.89 ^n^
CHX (0.12%)	11.00 ± 2.00^a^	3.83 ± 1.47 ^c^	23.50 ± 0.84^d^	19.33 ± 0.82 ^g^	9.83 ± 1.47 ^j^	1.16 ± 0.98 ^m^
NaF (1000 ppm)	11.00 ± 1.10^a^	3.00 ± 1.10 ^c^	21.33 ± 1.03^e^	16.17 ± 1.17 ^h^	5.83 ± 1.17^k^	0.00 ± 0.00 ^m^

The letters E, Ds, Dm, and Dx represent enamel only, slightly dentinal, moderate dentinal, and extensive dentinal caries lesions, respectively. Values labelled with different superscript letters in the same column denote significant statistical differences among treatments (One-way ANOVA, p < 0.05).

After the 5-wk topical treatment, all rats maintained good physical health and no excess deaths or significant differences (p > 0.05) in weight gain were observed among the groups (Supplementary Figure 3A). In addition, histopathological analysis indicated no signs of harmful effects on the oral mucosa, including abnormal proliferation, inflammatory infiltration, and necrocytosis in oral soft tissues after treatment with LF-1 and LF-2 Supplementary Figure 3B).

## Discussion

Antimicrobial peptides (AMPs) have gradually been considered therapeutic alternatives to traditional antibacterial agents for the prevention and treatment of dental caries due to their broad antimicrobial spectrum, fast killing kinetics, low drug-resistance, and few adverse effects [7]. However, synthetic modified AMPs based on natural antimicrobial protein and peptide templates are preferred for the optimization of antibacterial activity for practical applications [13]. Considering that lactotransferrin is widely recognised as a caries-associated protein, we extracted one of its functional sequences and utilised a combination of synthesis strategies [38], including sequence modifications, de novo design, and template-assisted mimesis to design and obtain our experimental LF-1 and LF-2 peptides. To the best of our knowledge, both LF-1 and LF-2 demonstrated the most potent antibacterial and antibiofilm activity against dental caries among lactotransferrin and its derived peptides [17,39,40], corroborating the feasibility of our strategy regarding the rational extraction and modification of a functional sequence from existing caries-associated proteins and peptides.

Lactoferrampin was adopted as the model peptide because it not only possesses antibacterial activity, but also characteristic structural features for the design of novel AMPs [19]. Despite the common AMP feature of a highly positive charge and hydrophobic domain, the amphipathic N-terminal helix of lactoferrampin is relatively separated from the flexible cationic C-terminus instead of the typical alternating hydrophilic-hydrophobic arrangement [41]. We previously analysed the sequences of bovine and human lactoferrampin and found the N-terminus to be the most critical functional sequence responsible for antibacterial activity [20], and the first seven amino acid residues crucial for the formation of the α-helix [42]. Further, a substitution of serine by arginine in position 5 was reported to enhance the net positive charge and improve the antibacterial activity of lactoferrampin [43]. Therefore, based on template-assisted and site-directed mutagenesis approaches, we extracted and modified the functional sequence, termed template sequence (WKLLRKA). We adopted a de novo strategy to fulfil the requirement for amphipathicity and enhance antimicrobial activity, and subsequently duplicated the template peptide sequence, obtaining LF-1 (WKLLRKAWKLLRKA). Furthermore, N-terminal helix cap residues (GKLI) were shown to stabilise the α-helical structure and enhance the mediation of membrane insertion, thus increasing the antibacterial activity [43]. Consequently, we added the helix cap motif to the N-terminus of LF-1, obtaining LF-2 (GKLIWKLLRKAWKLLRKA) (Supplementary Figure 4).

Both the net positive charge and amphipathicity, which enable AMPs to adsorb to the anionic microbial surface and disrupt the microbial membrane through the pore formation mechanism, are prerequisites for the antibacterial activity of α-helical AMPs [44]. Compared with the parent peptide lactoferrampin, both LF-1 and LF-2 showed perfect amphiphilic arrangement and strong net charge under a physiological environment (Table 1 and Figure 1). Considering that the autofluorescence of tryptophan residues is sensitive in a hydrophobic environment [45], the tryptophan fluorescence spectra indicated that both peptides could insert more deeply into the hydrophobic core of the mimetic membranes than lactoferrampin. Taken together, these structural features resulted in both LF-1 and LF-2 acquiring outstanding antibacterial activity against caries-associated bacteria compared with lactoferrampin. Notably, given that the helix cap motif stabilised the secondary structure of LF-2, LF-2 displayed a better amphiphilic structure, more substantial net charge, higher α-helical content, and more intense tryptophan fluorescence than LF-1, corroborating the improved antibacterial activity of LF-2.

It is worth noting that LF-1 expressed selective antibacterial activity, targeting *S. mutans* and *Actinomyces* species with relative lower MIC values, while *Lactobacillus* species and other oral commensal bacteria appeared to be more resistant to LF-1. Despite their characterization as cariogenic bacteria, *Lactobacillus* species are now increasingly being considered probiotics in the oral cavity [46]. The difference in MICs suggested that LF-1 might possess a selective capacity to suppress cariogenic bacteria but preserve beneficial commensal bacteria. If used to prevent and treat dental caries, the potential resistance of *Lactobacillus* species and commensal bacteria to LF-1 might minimise the disturbance to the normal microbiota, thus avoiding an imbalance of the oral ecosystem [5].

Oral bacteria in a biofilm state have often been associated with dental plaque, with the highly acidogenic *S. mutans* biofilm often being considered the most important pathogenic factor for dental caries [47]. Thanks to their rapid bactericidal mechanism, both peptides could effectively eradicate *S. mutans* in the preformed biofilms at low concentrations (32 and 64 μM). Hence, we adopted these peptide concentrations in the subsequent animal experiment. Furthermore, our *in vivo* results indicated that both LF-1 and LF-2 could significantly reduce the incidence of smooth-surface and sulcal-surface caries compared with the negative control. Both peptides displayed powerful anticaries effectiveness *in vivo* rivalling that of NaF and CHX, which are considered regular anticaries agents. Hence, the potent antibiofilm and anticaries activities of these peptides suggested their broad clinical application prospects.

Interestingly, LF-1 and LF-2 exhibited different haemolytic toxicities, with LF-1 demonstrating much lower haemolytic toxicity than LF-2, even at a high concentration. Although the α-helical structure with an amphiphilic interface is essential for the antibacterial activity of AMPs, the promotion or stabilisation of the hydrophobic surface is known to induce haemolytic toxicity [48]. Therefore, completely overcoming cytotoxicity while improving the antibacterial activity of AMPs is difficult, and thus the goal is to minimise cytotoxicity as much as possible. Nevertheless, our animal model results indicated that both LF-1 and LF-2 possessed low local toxicity and satisfactory biocompatibility because the oral soft tissues of rats did not exhibit histopathological changes after topical use, and no adverse effects were observed on the health of animals. Considering that AMPs are generally used topically to combat dental caries, the cytotoxicity of LF-2 was deemed acceptable [49].

Finally, the limitations and prospects of the study should be addressed. Considering that dental biofilm is an important ecosystem in the oral cavity with a complex spatial structure and microbial composition, we should adopt a more sophisticated caries model of a biofilm composed of multiple cariogenic bacteria in a future study [50]. Furthermore, the species diversity of the oral microbiota, especially the presence of probiotics and commensal bacteria, can maintain the ecological balance of the oral cavity and reverse the transition to oral diseases [51]. Therefore, the selective inhibition of cariogenic bacteria by LF-1 and its ecological impacts on the composition of the oral microbiota should be further confirmed and investigated [52]. Although the antibacterial activity of our peptides was attributed to the transmembrane pore formation mechanism, future studies should focus on the intracellular targeting and immunomodulation mechanisms [53]. Additionally, the relatively enhanced haemolytic toxicity of LF-2 is of concern despite its favourable biocompatibility in the rodent model. This issue might be addressed by appropriately adjusting the LF-2 sequence or by introducing positively charged residues to balance the antibacterial activity with cytotoxicity [48]. To date, a series of clinical application studies have investigated AMPs combating dental caries [54,55], and we should identify future clinical application possibilities for these peptides in the composite-tooth interface, the coating of tooth surfaces, and oral hygiene products.

## Conclusions

In conclusion, we successfully designed and synthesised novel AMPs, termed LF-1 and LF-2, based on the functional sequence of lactotransferrin. Both peptides possessed good amphipathicity and a strong capacity to form an α-helical structure. Due to their characteristic structural properties, both peptides exerted potent activity against planktonic cariogenic bacteria, inhibited the formation of *S. mutans* biofilm, demonstrated acceptable haemolytic activity, and induced notable preventive effects on dental caries *in vivo*. Hence, our null hypothesis was rejected. Based on the functional sequence of existing caries-associated proteins and peptides, our results provided two novel AMPs as promising anticaries agents and an approach to expand the scope of AMPs in treating dental caries. However, a balance between their antibacterial properties and haemolytic toxicity might be essential for the future application of antimicrobial peptides. Notably, the potential selective inhibition of planktonic cariogenic bacteria highlighted LF-1 as a promising candidate for the prevention and treatment of dental caries.

## Supplementary Material

Supplemental Material
