# Supplementary material for: Novel lactotransferrin-derived synthetic peptides suppress cariogenic bacteria in vitro and arrest dental caries in vivo: [Novel lactotransferrin-derived anticaries peptides]
Source: J Oral Microbiol. 2021 Jun 20;13(1):1943999. doi: 10.1080/20002297.2021.1943999 (PMC8216265; doi:10.1080/20002297.2021.1943999)
Supplement: Supplemental Material [file ZJOM_A_1943999_SM2061.zip › Supplementray/Supplementary document 1.docx]

**Stability test for LF-1 and LF-2**

**Materials and Methods**

The stability of LF-1 and LF-2 in conventional solvents, including DDW, PBS and HEPES, were determined as described with some modifications [1]. Both peptides were dissolved in DDW, 10 mM PBS (pH 7.4) and 10 mM HEPES buffer (containing 100 mM NaCl, pH 7.4) to a final concentration of 64 μmol/L. These solutions were incubated at 37 °C, and samples collected at 0, 1, 3, 6, 12, 24 h were subjected to Ultra Performance Liquid Chromatography (UPLC; Acquity, Waters, Milford, MA, USA) for analysis. We obtained the chromatograms of peptides in different solutions at different time points. The peak area of peptides at each time point was calculated and compared with that of the positive control (at 0 h) to determine the retention rates of peptides. The data were obtained from 3 independent experiments, and statistical analysis was conducted via one-way analysis of variance (ANOVA).

**Results**

*LF-1 and LF-2 displayed favourable stability in the conventional solutions*

As shown in Figure R1, both the peptides were stable in these conventional solutions up to 24 h at 37 °C. LF-1 expressed slight degradation of less than 5 %, while LF-2 kept mostly stable as time went by. There was no significant difference in the retention rates of peptides in different solutions at each time point (p > 0.05). The retention rates of LF-1 and LF-2 could maintain over 95 % and 98 %, respectively, for 24 h at 37 °C. These results revealed that both LF-1 and LF-2 possessed good stability in the conventional solutions.





**Figure R1.** Stability analysis of LF-1 and LF-2 dissolved in DDW, PBS, and HEPES and incubated up to 24 h at 37 °C. Calculation is based on the peak area of peptides in the chromatograms, and the retention rate of peptides is represented as mean ± SD and plotted at each time point.

**Reference**

[1] R.K. Thapa, H.C. Winther-Larsen, D.B. Diep, H.H. Tonnesen, Preformulation studies on novel garvicin KS peptides for topical applications, Eur J Pharm Sci 151 (2020) 105333. https://doi.org/10.1016/j.ejps.2020.105333
