# Supplementary figures and images for: Novel lactotransferrin-derived synthetic peptides suppress cariogenic bacteria in vitro and arrest dental caries in vivo: [Novel lactotransferrin-derived anticaries peptides]
Source: J Oral Microbiol. 2021 Jun 20;13(1):1943999. doi: 10.1080/20002297.2021.1943999 (PMC8216265; doi:10.1080/20002297.2021.1943999)

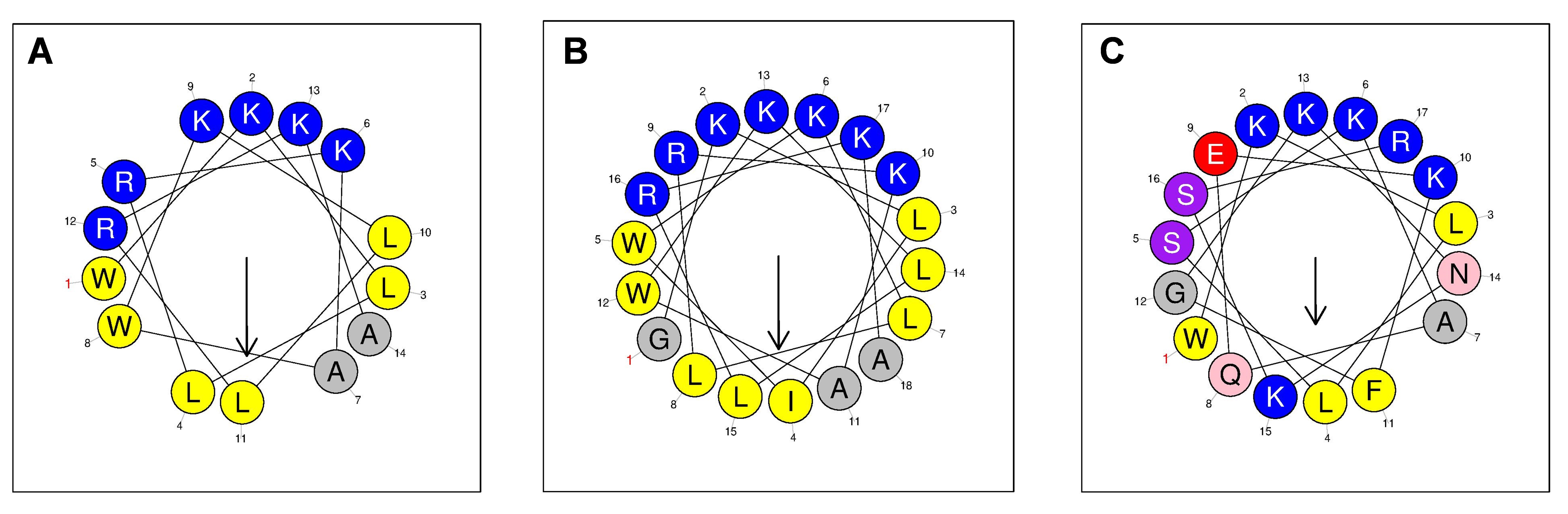

Supplement: Supplemental Material [file ZJOM_A_1943999_SM2061.zip › Supplementray/Supplementary Figure 1.jpg]

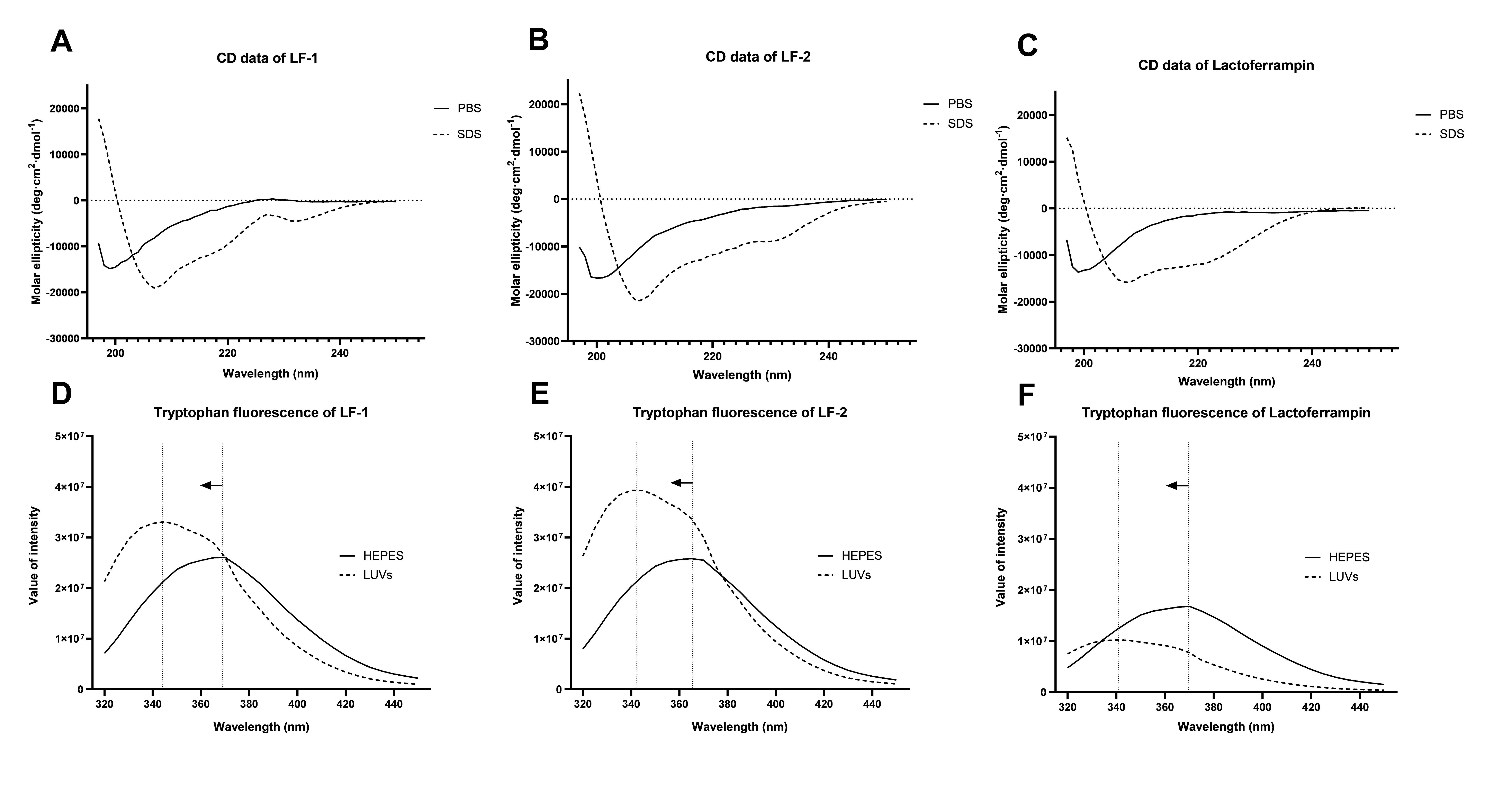

Supplement: Supplemental Material [file ZJOM_A_1943999_SM2061.zip › Supplementray/Supplementary Figure 2.jpg]

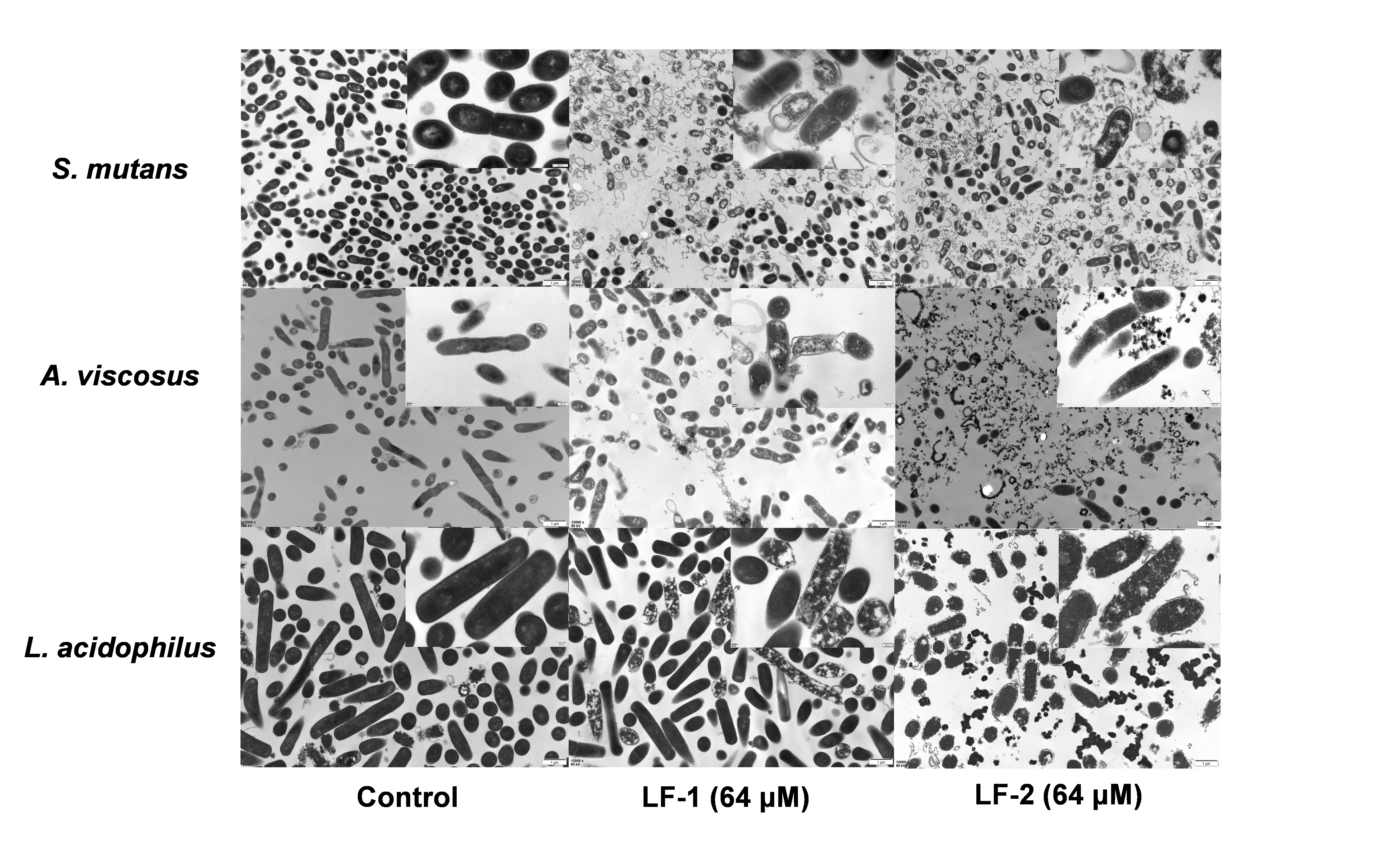

Supplement: Supplemental Material [file ZJOM_A_1943999_SM2061.zip › Supplementray/Supplementary Figure 3.jpg]

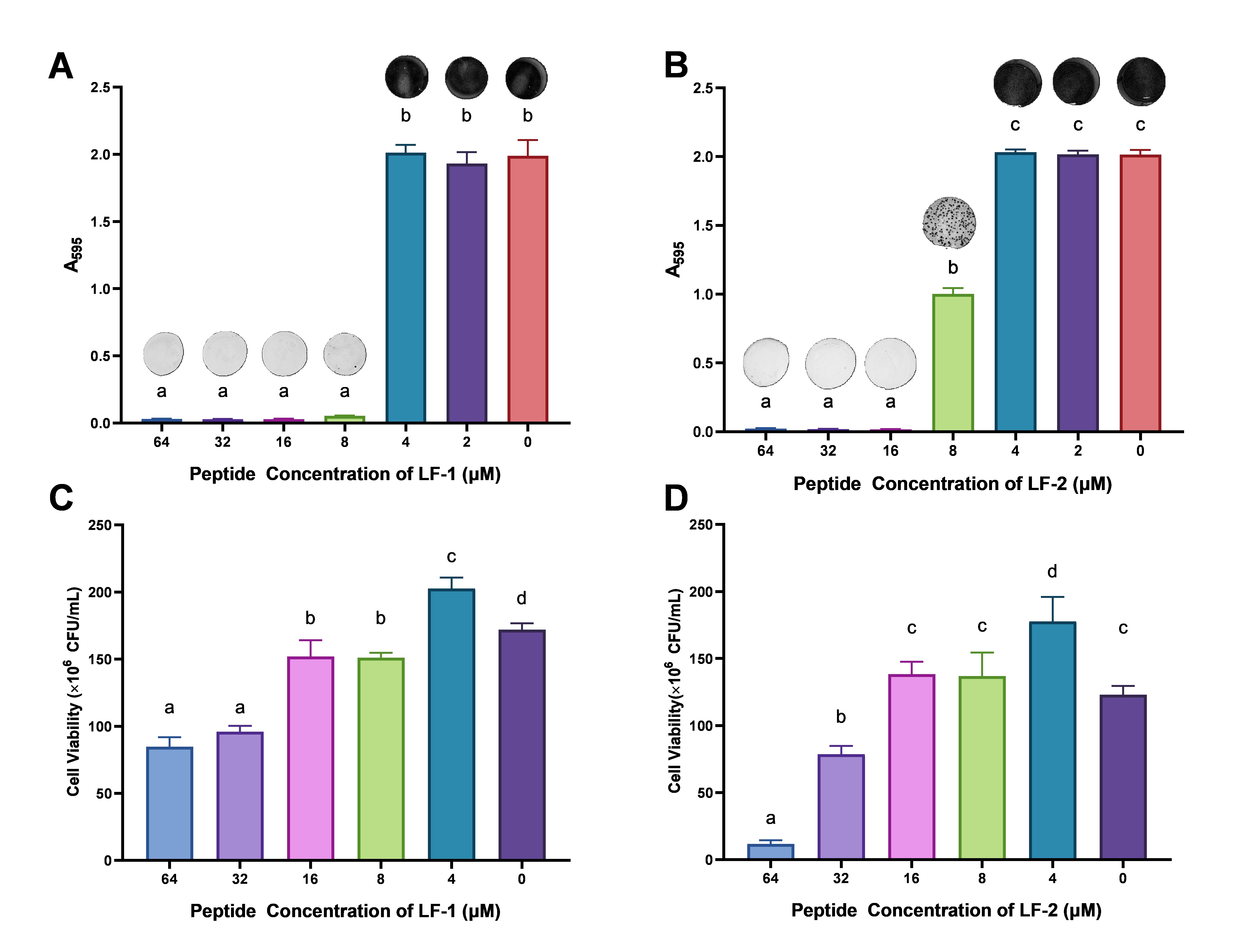

Supplement: Supplemental Material [file ZJOM_A_1943999_SM2061.zip › Supplementray/Supplementary Figure 4.jpg]
